# Laser-Driven Surface Alloying of Ti6Al4V: Coupled Microstructural Evolution, Phase Behavior, and Mechanical Performance

**DOI:** 10.3390/ma18184237

**Published:** 2025-09-09

**Authors:** Hana Beyene Mamo, Klaudiusz Gołombek, Gilmar Ferreira Batalha, Marcin Adamiak

**Affiliations:** 1 Faculty of Mechanical Engineering, Silesian University of Technology, Konarskiego Street 18A, 44-100 Gliwice, Poland; klaudiusz.golombek@polsl.pl; 2 Department of Mechatronics and Mechanical Systems Engineering, Polytechnic School of Engineering of the University of Sao Paulo (USP), Sao Paulo 05508-900, Brazil; gfbatalh@usp.br

**Keywords:** Ti6Al4V, laser surface alloying, silver (Ag), copper (Cu), microstructures

## Abstract

This study investigates the microstructural and mechanical evolution of Ti6Al4V alloy surfaces modified through laser surface alloying (LSA) with antimicrobial elements silver (Ag) and copper (Cu) to enhance surface performance for biomedical applications. The as-received Ti6Al4V exhibited a typical equiaxed α-β microstructure with baseline hardness. Following LSA treatment using a 1000 W pulsed laser, distinct transformations were observed in the melt zone (MZ) and heat-affected zone (HAZ), influenced by the specific alloying element. Ag incorporation led to the development of ultrafine acicular martensitic structures and a higher fraction of high-angle grain boundaries, resulting in moderate hardness improvement. In contrast, Cu alloying promoted the formation of Ti_2_Cu intermetallic phases, dendritic morphologies, and pronounced solute segregation, leading to a more significant increase in hardness. Electron Backscatter Diffraction(EBSD) and Energy Dispersive Spectroscopy (EDS) analyses revealed grain refinement, texture evolution, and elemental redistribution across the modified regions, while X-ray Diffraction XRD confirmed the presence of new phases. The comparative analysis highlights that although both Ag and Cu improve microstructural complexity and hardness, Cu-modified zones exhibited higher hardness values than Ag-modified zones, suggesting a stronger surface strengthening effect under the tested conditions. These findings contribute valuable insights into the structure–property relationships of LSA-modified Ti alloys, supporting their potential for durable and antimicrobial biomedical implants.

## 1. Introduction

Ti6Al4V titanium alloy (Grade 5) is one of the most widely used biomaterials for orthopedic and dental implants because of its unique combination of low density, high specific strength, excellent corrosion resistance, and superior biocompatibility [1,2,3,4]. Compared with other metallic biomaterials, such as stainless steel and cobalt–chromium (Co–Cr) alloys, Ti6Al4V provides significant advantages for long-term implantation [5]. Stainless steel, while inexpensive, suffers from poor corrosion resistance and nickel ion release, which can cause allergic and inflammatory reactions [6,7]. Co–Cr alloys, though mechanically strong and more wear-resistant, are denser and less biocompatible, with concerns about the toxic effects of cobalt and chromium ion release [8,9]. In contrast, Ti6Al4V combines mechanical reliability with excellent biocompatibility, making it the most widely accepted material for permanent load-bearing implant [10,11,12].

Despite its widespread clinical use, a critical limitation of Ti6Al4V is the absence of inherent antibacterial activity. This shortcoming renders the alloy highly susceptible to bacterial adhesion and subsequent biofilm formation, which not only elevates the risk of implant-associated infections but can also result in severe complications, including implant failure [13,14,15,16]. Evidence from recent in vitro studies confirms that Ti6Al4V surfaces are rapidly colonized by pathogenic bacteria such as Staphylococcus aureus and Staphylococcus epidermidis, with mature biofilms forming within a matter of hours [17,18]. Clinical findings reinforce these observations: periprosthetic joint infections account for 0.5–2.3% of orthopedic implant cases, representing one of the leading causes of revision surgery and often necessitating complex, high-cost interventions [19,20]. Similarly, in dental applications, infection rates have been reported at 9.3–12.8% per implant and 18.5–19.8% per patient [21,22].

Scientific investigations attribute this vulnerability to the physicochemical interaction between the alloy surface and its biological environment. The passive TiO_2_ film rapidly binds host proteins, forming a conditioning layer that facilitates bacterial adhesion. Surface properties such as wettability, nanoscale roughness, and the absence of antibacterial ion release further enhance bacterial persistence [23,24,25,26].

To overcome the lack of inherent antibacterial activity in Ti6Al4V, considerable research has focused on incorporating antibacterial elements most notably silver (Ag) and copper (Cu) as effective strategies to enhance antimicrobial performance [27,28,29,30,31]. Ren et al. [32] investigated Ti6Al4V–xCu (x = 1, 3, 5 wt%) alloys and reported a progressive improvement in antibacterial efficacy with increasing Cu content. Complementary findings were reported by Liu et al. [33], who developed a novel Ti–Cu alloy for dental implants and demonstrated strong antibacterial activity against Streptococcus mutans and Porphyromonas gingivalis. Similarly, Lei et al. [34] confirmed that the addition of Ag to Ti6Al4V alloys effectively suppressed bacterial adhesion and biofilm formation, thereby improving overall antibacterial performance.

Recent investigations have extended these strategies through laser surface alloying (LSA), a technique that provides precise control over alloy composition, microstructural evolution, and resulting mechanical performance [35,36,37,38]. In particular, Cu-alloyed Ti6Al4V produced by LSA has demonstrated long-term antibacterial activity while also exhibiting enhanced wear resistance, a result attributed to the formation of refined microstructures [28,29]. Similarly, Ag-modified Ti6Al4V surfaces prepared via LSA have shown strong antibacterial efficacy against Staphylococcus aureus and Escherichia coli, owing to the controlled release of Ag ions, while retaining excellent cytocompatibility with osteoblast-like cells [27,34]. Beyond their antimicrobial functions, both Ag and Cu modifications achieved through LSA contribute to improved corrosion resistance in simulated physiological environments by stabilizing the passive oxide film and mitigating localized corrosion [30,31]. Collectively, these findings highlight that LSA not only overcomes the absence of intrinsic antibacterial functionality in Ti6Al4V but also provides a versatile means of tailoring its mechanical, corrosion, and biological properties [39,40,41,42].

Despite notable progress in laser surface alloying (LSA) of Ti6Al4V with antibacterial elements such as Ag and Cu, the fundamental microstructural transformations within the molten and heat-affected regions remain insufficiently understood. In particular, key features including phase composition, grain size and morphology, and grain boundary characteristics have not been systematically investigated, even though each of these parameters plays a critical role in governing both the antibacterial efficacy and mechanical performance of the alloy [28,43,44].

To address these gaps, the present study employs a 1000 W pulsed laser to alloy silver (Ag) and copper (Cu) onto the surface of Ti6Al4V and systematically investigates the resulting process–structure–property relationships. Particular attention is given to the microstructural evolution within the melt and heat-affected regions and its correlation with mechanical behavior. A combination of advanced characterization techniques, including Scanning Electron Microscopy (SEM), XRD, Electron Channeling Contrast (ECC) imaging, EBSD, and EDS, is utilized to examine grain morphology, phase composition, and local chemistry. Beyond conventional analysis, EBSD data are processed using Python-based tools to statistically evaluate grain size distributions and grain boundary misorientation, while EDS derived compositions are employed in Scheil solidification simulations to predict phase evolution under rapid cooling. Microhardness measurements are further correlated with these experimental and computational findings, enabling a comprehensive understanding of how Ag and Cu incorporation via LSA influences the mechanical related potential of Ti6Al4V.

## 2. Materials and Methods

The as-received material used in this study was a Ti6Al4V alloy plate, cold-rolled and subsequently recrystallization-annealed. Its chemical composition (wt%) was Ti 92.21, Al 5.66, and V 2.13. The β-transus was approximately 980 °C, and the melting range (solidus–liquidus) spanned 1604–1660 °C [45].

**Figure 1:**
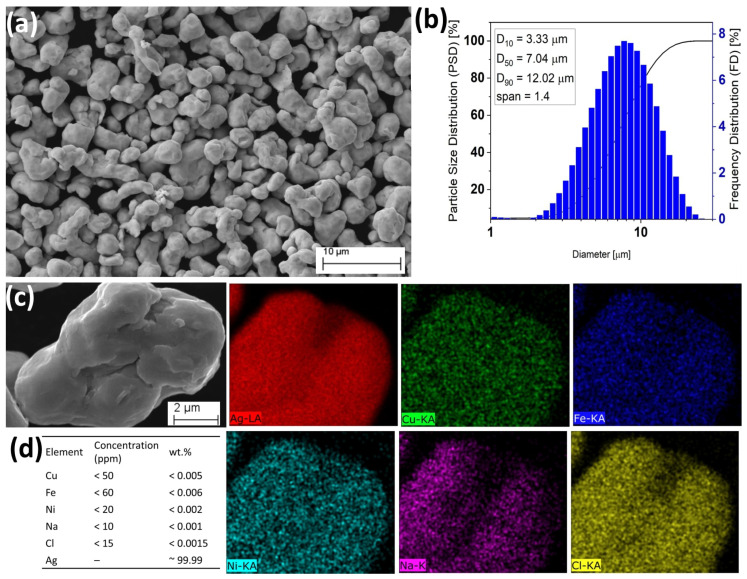
Silver (Ag) powder characterization. (**a**) SEM micrograph of powder morphology, (**b**) powder particle size distribution plot showing statistical derivations and computed particle span, and (**c**) energy dispersive X-ray spectroscopy (EDS) maps showing the chemical compositions of the Ag powder particles. (**d**) Manufacturer-reported impurity levels for the Ag powder is 99.99%.

**Figure 2:**
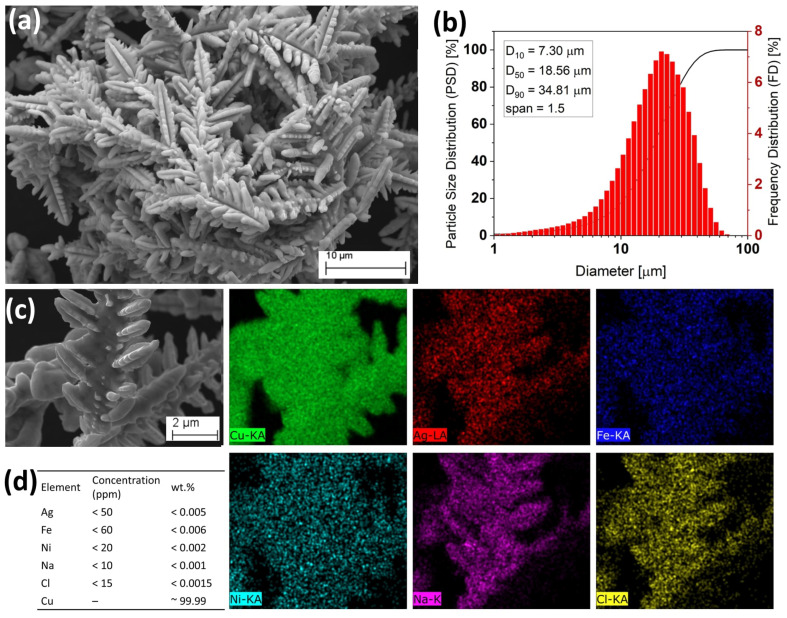
Copper (Cu) powder characterization. (**a**) SEM micrograph of powder morphology, (**b**) powder particle size distribution plot showing statistical derivations and computed particle span, and (**c**) energy dispersive X-ray spectroscopy (EDS) maps showing the chemical compositions of the Cu powder particles. (**d**) Manufacturer-reported impurity levels for the Cu powder is 99.99%.

Silver (Ag) and copper (Cu) powders, each with a density ≥10.59 g/cm^3^, were procured from NMD New Materials Development GmbH and employed as alloying agents. The particle size distribution was determined using a laser particle size analyzer (ANALYSETTE 22, Fritsch, NJ, USA), while particle morphology and microstructural features were examined with a Zeiss EVO MA 15 scanning electron microscope (Carl Zeiss AG, Oberkochen, Germany). The Ag powder (Figure 1) exhibited predominantly irregular particles with a fraction of spherical counterparts, with size analysis yielding $d_{10}$ = 3.33 µm, $d_{50}$ = 7.04 µm, and $d_{90}$ = 12.92 µm. The calculated span of 1.4 indicated a high degree of particle uniformity, favorable for laser surface alloying. EDS mapping confirmed Ag as the major element, with trace levels of Cu, Fe, Ni, Na, and Cl, while the manufacturer (New Materials Development (NMD), Rosenheim, Germany) reported a purity of 99.99%. Similarly, the Cu powder (Figure 2) revealed irregular but smooth-surfaced particles, with $d_{10}$ = 7.30 µm, $d_{50}$ = 18.56 µm, and $d_{90}$ = 34.81 µm, corresponding to a span of 1.5, again suggesting uniformity. EDS analysis showed Cu as the dominant element with minor traces of Ag, Fe, Ni, Na, and Cl, and the manufacturer-specified purity was 99.99%. These results confirm that both Ag and Cu powders possessed high purity, favorable uniformity, and suitable size distributions, making them appropriate for the subsequent laser surface alloying process.

For surface pre-treatment, a slurry of Ag or Cu powder dispersed in a 4 wt% polyvinyl alcohol (PVA) aqueous solution was uniformly applied onto the rolling–transverse (RD–TD) surfaces of the specimens using a brush, forming a consistent precursor layer that was left to dry for 20 min under ambient conditions. Subsequent laser surface alloying (LSA) was carried out using a TRUMPF TruDisk 3302 disk laser (TRUMPF, Ditzingen, Germany) operating in continuous-wave (CW) mode, capable of delivering up to 3.3 kW of stable output power for uniform melting and alloying. Processing was performed at a scanning speed of 200 mm/s along the transverse direction with a beam power of 1000 W, providing sufficient energy input for localized melting of the Ag/Cu slurry-coated surface. The laser beam was focused to a spot diameter of approximately 245 µm, with the focal point positioned 20 mm above the substrate to maintain a consistent energy density. Each specimen was treated with a single laser pass to minimize heat accumulation and preserve substrate integrity, while continuous argon shielding at a flow rate of 15 L/min was supplied to the irradiated zone to prevent oxidation during processing.

**Figure 3:**
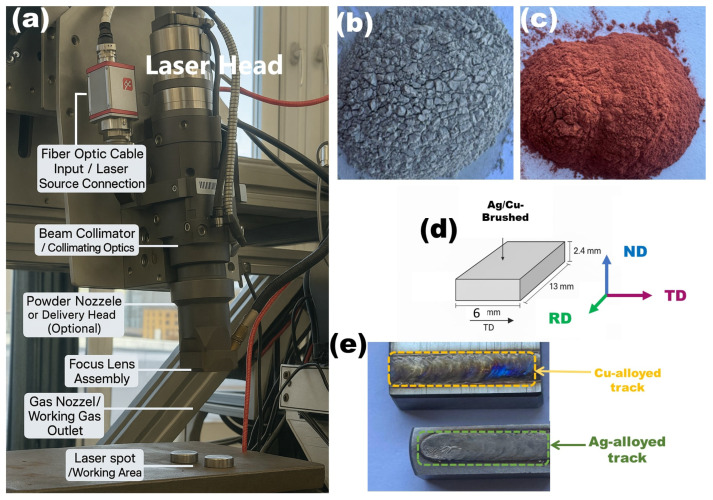
Schematic and photographic overview of the LSA workflow. (**a**) The laser processing head showing key components. (**b**) Ag powder used for surface alloying. (**c**) Cu powder used for surface alloying. (**d**) Sample geometry and reference directions (RD/TD/ND). (**e**) Representative Cu- and Ag-alloyed tracks after LSA.

Specimens were sectioned from the Ti6Al4V plate with dimensions of 6 mm (TD), 13 mm (RD), and 2.4 mm (ND), as illustrated in Figure 3. Microstructural characterization was performed on the RD–ND planes, oriented perpendicular to the laser beam travel direction. Prior to analysis, samples were sequentially ground using SiC abrasive papers, followed by intermediate polishing with diamond suspensions and final polishing with colloidal silica on a TEGRAMIN 30 automated system (Struers A/S, Ballerup, Denmark), yielding flat, scratch-free surfaces suitable for SEM, EBSD, and XRD investigations. To enhance the visibility of microstructural features, polished specimens were etched in Keller’s reagent (distilled water, nitric acid, hydrochloric acid, and hydrofluoric acid) for 20 s. Surface morphology and microstructural features were then examined using a Zeiss EVO MA 15 scanning electron microscope (Carl Zeiss AG, Oberkochen, Germany) equipped with an energy-dispersive X-ray spectrometer (EDS), while phase analysis was carried out using a Bruker D8 Advance diffractometer (Bruker AXS GmbH, Karlsruhe, Germany).

Rectangular coupons (6 mm (TD) × 13 mm (RD) × 2.4 mm (ND)) were sectioned from the Ti6Al4V plate (Figure 3). Microstructural characterization was performed on the RD–ND plane, oriented perpendicular to the laser scan direction. Specimens were sequentially ground with SiC abrasive papers, followed by intermediate diamond polishing and a final colloidal–silica polish on a TEGRAMIN 30 automated system (Struers A/S, Ballerup, Denmark) to obtain flat, scratch-free surfaces suitable for SEM, EBSD, and XRD. EBSD was conducted on the as-polished surface using a Zeiss EVO MA 15 scanning electron microscope (Carl Zeiss AG, Oberkochen, Germany) operated at an accelerating voltage of 20 kV, a working distance of 15 mm, and a step size of 0.10 µm. For enhanced contrast in conventional SEM imaging, selected specimens were subsequently etched in Keller’s reagent (distilled water, nitric acid, hydrochloric acid, and hydrofluoric acid) for 20 s. Surface morphology and EDS analyses were performed on the same SEM, and phase identification by XRD employed a Bruker D8 Advance diffractometer (Bruker AXS GmbH, Karlsruhe, Germany).

Microhardness measurements were conducted using a Walter Uhl microhardness tester (Walter Uhl Technische Mikroskopie GmbH & Co. KG, Asslar, Germany) in accordance with the ASTM E384 standard [46] for microindentation hardness testing. A load of 500 g was applied, and indentations were introduced along a straight line perpendicular to the laser beam travel direction on the RD–ND surface. For each specimen, ten indentations were made at intervals of 100 µm, and the average of these values was reported as the representative hardness. Data processing and statistical analysis were carried out using Python 3.10 (Python Software Foundation, Wilmington, DE, USA). In addition, chemical composition data obtained from EDS measurements of the melt region were used as input for Scheil solidification simulations performed with Thermo-Calc software (version 6, Thermo-Calc Software AB, Stockholm, Sweden) to evaluate phase evolution under rapid solidification conditions.

## 3. Results and Discussion

### 3.1. Initial State of Ti6Al4V Alloy

**Figure 4:**
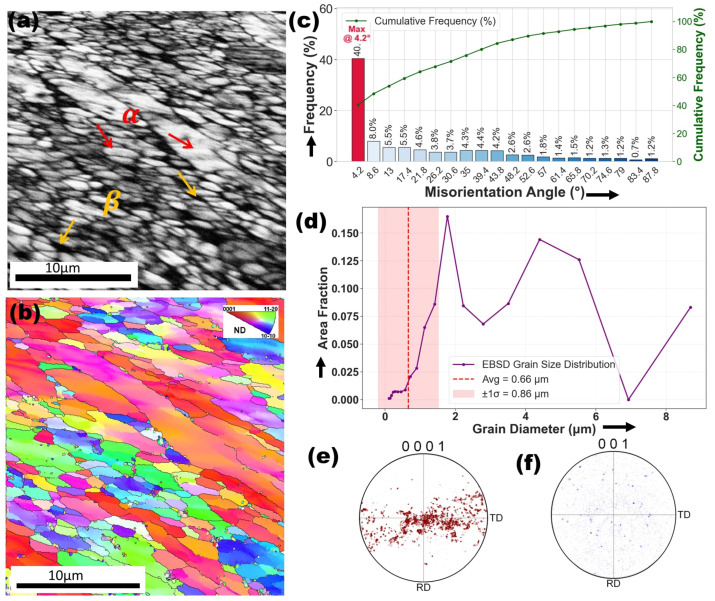
Microstructural and crystallographic analysis of the as-received Ti6Al4V alloy. (**a**) BCC micrograph showing the grain morphology. (**b**) EBSD inverse pole figure (IPF) map illustrating grain orientation. (**c**) Grain boundary misorientation angle distribution. (**d**) Grain size distribution plot. (**e**,**f**) Pole figures showing the crystallographic texture of the α-phase (0001) and β-phase (001) along the rolling direction (RD) and transverse direction (TD).

Figure 4a presents an ECC image depicting the microstructure of the as-received specimen. The image reveals two distinct morphologies: nearly equiaxed grains and uniformly distributed short-rod-shaped second-phase particles. These features correspond to α-Ti and β-Ti, respectively. The α phase exhibits a hexagonal close-packed (hcp) structure, while the β phase has a body-centered cubic (bcc) structure, as previously reported in the literature [47]. The volume fractions of the α and β phases were found to be approximately 96.7% and 3.3%, respectively. Figure 4b presents an EBSD inverse pole figure (IPF) map, showing a significant presence of subgrains delineated by low-angle boundaries (LABs, $2^{\circ }<\theta <15^{\circ }$) within the α matrix, along with fully recrystallized grains enclosed by high-angle boundaries $\left(HABs,\theta >15^{\circ }\right)$. Based on the standard triangle in Figure 4b, the subgrains exhibit a preferred orientation of $\langle 11\bar{2}0\rangle$ along the normal direction (ND). In contrast, the c-axes of most recrystallized grains are predominantly aligned toward the transverse direction (TD). The misorientation angle distribution (MAD) histogram associated with Figure 4b is presented in Figure 4c indicating that the majority of misorientation angles are below $15^{\circ }$. A high fraction of LABs, peaking at $4.2^{\circ }\;\left(0.40\right)$, with additional peaks at $8.6^{\circ }\;\left(0.08\right)$ and $13^{\circ }\;\left(0.05\right)$, indicating sub-grain structures formed due to plastic deformation or partial recrystallization. HABs become more pronounced at higher angles but remain less frequent, with peaks at $17.4^{\circ }\;\left(0.05\right)$, $30.6^{\circ }\;\left(0.03\right)$, and $39.4^{\circ }\;\left(0.04\right)$, signifying localized dynamic recrystallization. The grain size distribution (Figure 4d) reveals a fine-grained microstructure with a mean grain diameter of 0.66 µm. The dominant size range is 1.41–2.79 µm, while finer grains (∼0.11–0.2 µm) have low area fractions. Larger grains (>5 µm) are rare, except for an 8.69 µm grain (0.08 area fraction). The high standard deviation (0.86 µm) indicates a heterogeneous grain size distribution, suggesting partial recrystallization. The (0001) pole figure for the hcp α-phase (Figure 4e) exhibits clustering along the RD-TD directions, indicating a preferred texture influenced by rolling in the as-received specimen [48]. In contrast, the (001) PF for the β-phase (Figure 4f) shows a scattered distribution, suggesting a weak or random texture characteristic of retained β-phase following rapid solidification or phase transformation [49].

### 3.2. Microstructures of the LSAed Specimens

**Figure 5:**
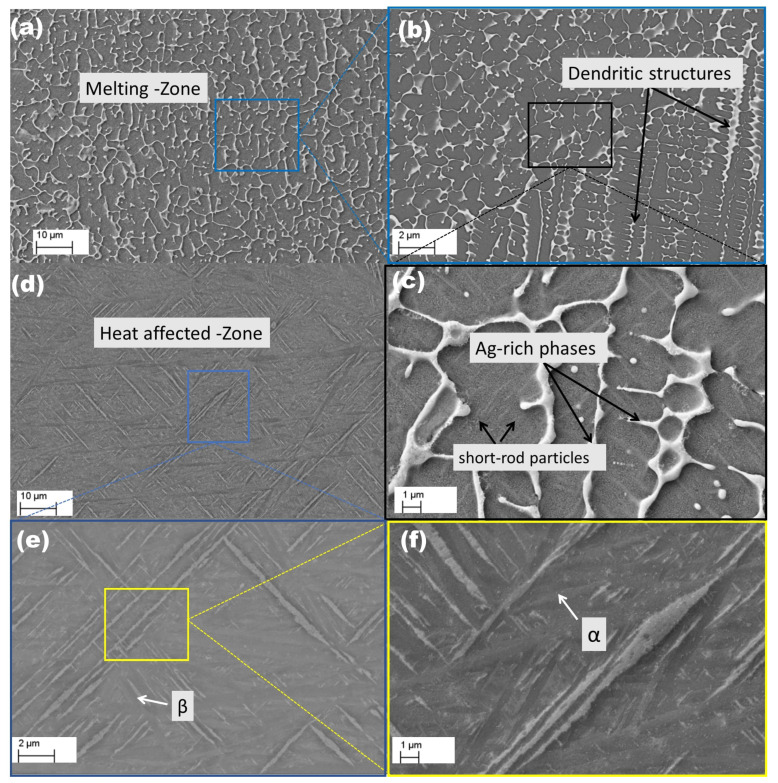
SEM micrographs of Ti6Al4V alloyed with silver: (**a**–**c**) present the microstructural features within the melting region, whereas (**d**–**f**) illustrate the morphological characteristics of the heat-affected zone.

Figure 5 presents an SEM micrograph depicting the microstructural changes in the Ti6Al4V alloy after laser surface alloying with silver (Ag). Figure 5a shows a low-magnification SEM image of the melted zone (MZ) in the Ti6Al4V–Ag specimen, revealing a maximum height of approximately 290 µm and a width of around 740 µm. No microcracks or porosity were observed, suggesting that the high cooling rates and thermal stresses did not induce significant defects. A magnified view of a selected region in Figure 5b reveals a significant transformation in grain morphology within the melted zone (MZ) compared to the original microstructure. Some of the previously existing equiaxed α grains and short-rod β phases have been replaced by fine dendritic or cellular structures. A higher-magnification image of the melting region in Figure 5c shows that most dendritic or cellular structures exceed 1 µm in width. These densely interconnected dendritic structures are accompanied by distinct plate-like formations, indicative of a martensitic transformation within the region. Such transformations are commonly observed in titanium alloys subjected to rapid cooling during laser processing [50]. The presence of these plate-like structures suggests that the LSA induced a phase transition from the α→β martensite, driven by the high cooling rates involved [51]. Figure 5d presents the heat-affected zone (HAZ), with a height of approximately 110 µm. A clear distinction is observed between the microstructures of the HAZ and MZ, with the absence of dendritic structures in the HAZ. A further magnified image of the HAZ from Figure 5d, shown in Figure 5e, reveals two distinct microstructural features: short-rod particles dispersed throughout the matrix and fine plate-like structures. The short-rod particles correspond to the prior β-phase remnants from the initial microstructure, while the fine plates represent martensitic transformations originating from prior α grains through the α→β→α phase transition. Figure 5f is a significantly magnified image of the HAZ in Figure 5e, allowing microstructural features to be more clearly revealed.

**Figure 6:**
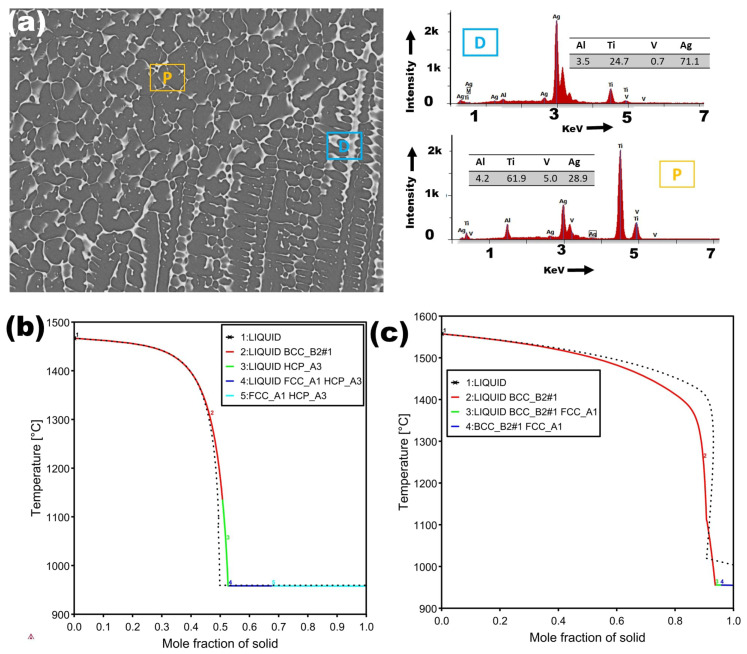
(**a**) SEM image of the laser surface alloyed Ti–6Al–4V–Ag sample with EDS spectra obtained from dendritic (D, blue) and interdendritic (P, yellow) regions. Region D is Ag-rich, whereas region P shows Ti-enriched phases. (**b**) Scheil solidification simulation of the D-region composition, predicting sequential phase evolution from liquid to Body-Centered Cubic (BCC_B2),Hexagonal Close-Packed (HCP_A3), and Face-Centered Cubic (FCC_A1) phases. (**c**) Scheil simulation of the P-region composition indicates extended liquid stability and final FCC (Ag) formation, consistent with the experimentally observed microsegregation.

Figure 6 illustrates the EDS analysis of the dendritic and plate-like structures in the melt zone (MZ) of the Ti6Al4V–Ag specimen, providing insight into their elemental distribution. It is evident that the dendritic structure (D) is enriched with Ag, whereas the plate structures (P) exhibit a higher concentration of Vanadium (V), a β-Ti stabilizer, relative to Aluminum (Al), α-Ti stabilizer.

**Figure 7:**
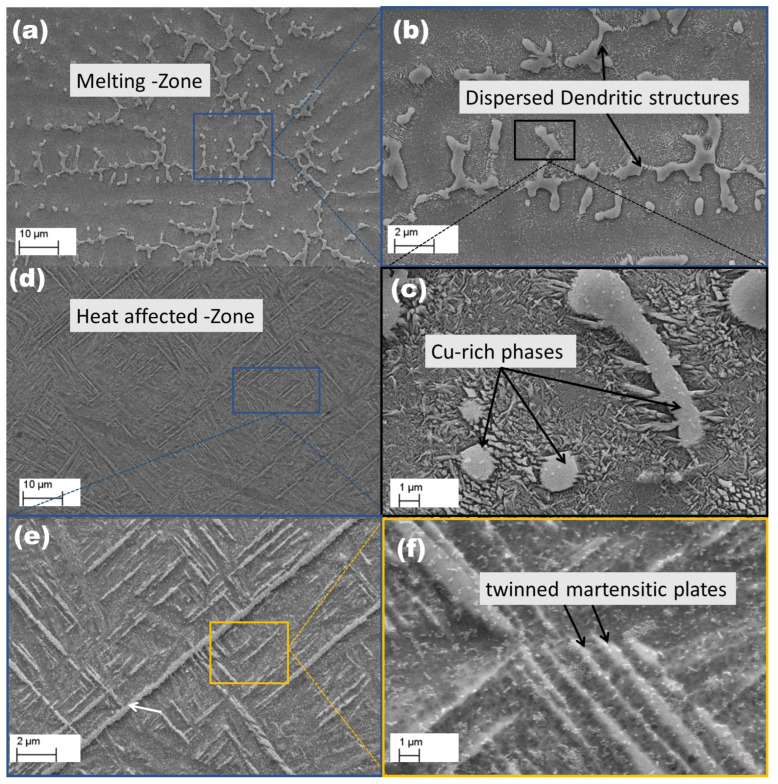
SEM micrographs of Ti6Al4V alloyed with copper: (**a**–**c**) display the melting region with dendritic structures; (**d**–**f**) illustrate the heat-affected zone with refined grain and lath structures.

Figure 7 presents cross-sectional SEM images of the Ti6Al4V–Cu specimen. Similar to the Ti6Al4V–Ag specimen, Figure 7a highlights the melting region of the Ti6Al4V–Cu sample. The melted zone extends to an estimated height of ∼300μm and a width of ∼820μm, which are larger compared to those observed in the Ti6Al4V–Ag specimen (Figure 5a). This variation is due to the distinct thermal and physical properties of Cu, as reported in previous studies [52]. Figure 7b,c provide magnified views of selected regions from Figure 7a,b, respectively, revealing that the MZ consists of dendritic structures similar to those observed in the Ti6Al4V–Ag specimen (Figure 5c). The primary distinction between the two lies in their elemental composition and the resulting microstructural characteristics. In the Ti6Al4V–Cu specimen, dispersed dendritic networks form, whereas in the Ti6Al4V–Ag specimen (Figure 5) they appear more interconnected. These differences suggest that the thermal and physical properties of Cu and Ag influence microstructural evolution during laser surface alloying. Figure 7e provides a magnified view of the Heat affected zone (HAZ) from Figure 7d, enhancing the distinction between different zones. The HAZ height is approximately ∼120μm, slightly surpassing the 110 μm observed in the Ti6Al4V–Ag specimen. A further magnified view of the HAZ is shown in Figure 7f, revealing a structure highly similar to that of the Ti6Al4V–Ag specimen but with a greater number of plate structures. The microstructural features of the Ti6Al4V–Cu HAZ closely resemble those of the Ti6Al4V–Ag specimen, exhibiting fine-twinned martensitic plates. However, Cu alloying introduces subtle variations, resulting in a slightly different acicular morphology characteristic of the α martensitic phase. These differences stem from the distinct thermal gradients and solidification behavior during processing. Figure 7f highlights the acicular morphology and fine-twinned martensitic plates in the Ti6Al4V–Cu specimen.

**Figure 8:**
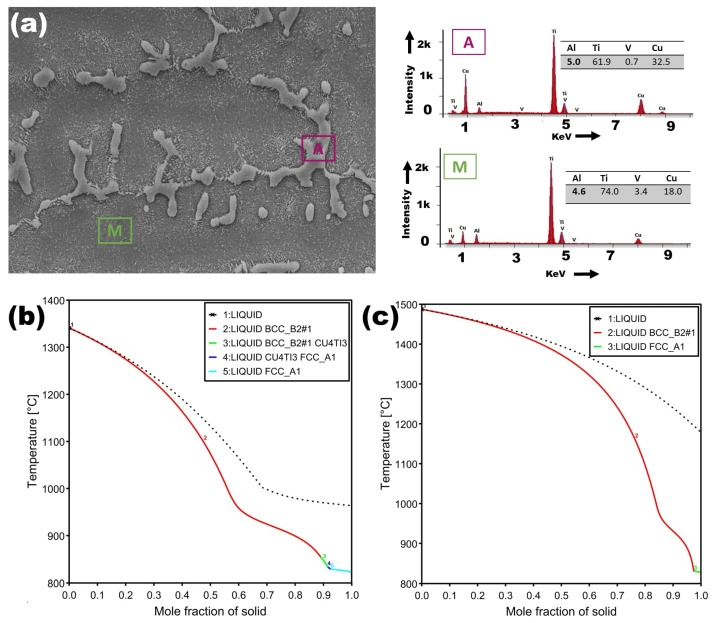
(**a**) SEM micrograph of the laser surface alloyed Ti6Al4V–Cu sample with EDS spectra obtained from the Cu-rich interdendritic region (A, purple) and the Ti-rich dendritic region (M, green). Region A shows ∼32.5 wt% Cu enrichment, whereas region M retains a lower Cu concentration of ∼18 wt%. (**b**) Scheil solidification simulation of the A-region composition, predicting sequential solidification from liquid to BCC_B2, Cu_4_Ti_3_, and FCC_A1 phases. (**c**) Scheil simulation for region M indicates dominant β-Ti solidification with limited segregation, consistent with the reduced Cu content observed by EDS.

Figure 8 presents the EDS analysis of the dispersed dendritic structure and matrix within the melting zone (MZ) of the Ti6Al4V–Cu specimen. Similar to Figure 6, the dendritic structure (A) exhibits a higher Cu content than the matrix (M), while the matrix (M) has a higher concentration of V. This indicates that the matrix structures in the MZ likely originate from prior α grain or subgrain transformations. The enrichment of Cu in the dendritic zones suggests phase segregation during solidification, leading to the possible formation of Ti-Cu intermetallic compounds, such as Ti_2_Cu.

**Figure 9:**
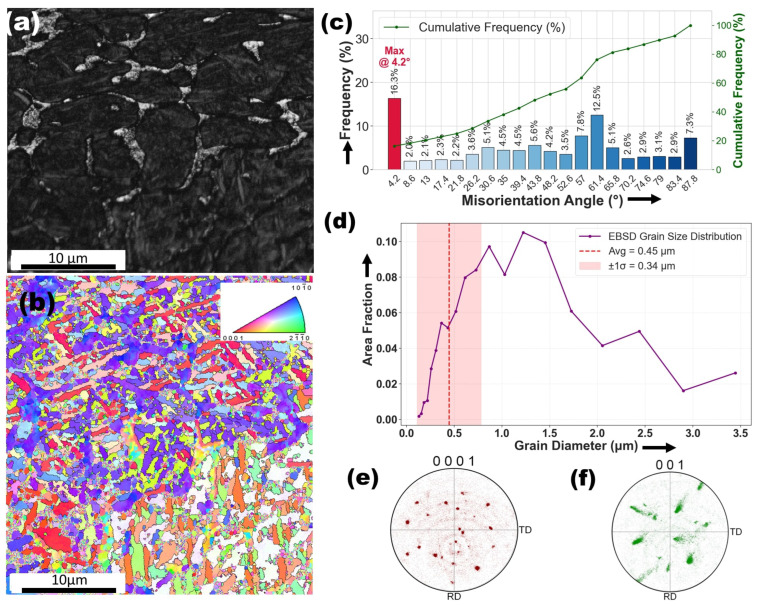
Microstructural and crystallographic analysis of the melting region of Ti6Al4V–Ag specimen after LSA. (**a**) BCC micrograph (**b**) EBSD inverse pole figure (IPF) map (**c**) misorientation angle distribution (**d**) grain size distribution plot. (**e**,**f**) Pole figures illustrating the crystallographic texture of the α-phase (0001) and β-phase (001) along the rolling direction (RD) and transverse direction (TD).

The EBSD analysis of the melted zone (MZ) in the Ti6Al4V–Ag specimen is illustrated in Figure 9. Figure 9a illustrates the band contrast (BC) map of the melting zone in the Ti6Al4V–Ag specimen, revealing finely dispersed plate-like structures aligned with the martensitic morphology noted in SEM observations. The corresponding inverse pole figure (IPF) map in Figure 9b shows a scattered grain orientation with no dominant texture, highlighting the randomized crystallographic arrangement induced by rapid solidification. These features differ substantially from the original microstructure in Figure 4a, where equiaxed grains and short-rod β-phase particles dominate, and Figure 4b, which displays a preferred crystallographic orientation and a significant presence of low-angle boundaries. The transformation is further evident in the higher fraction of high-angle grain boundaries and the refined grain sizes in the laser-treated region, signaling enhanced microstructural complexity and evolution. The dendritic and martensitic features in Figure 5a–c, along with the Ag-enriched dendrites and V-rich plates shown in Figure 6, further reflect the intense thermal gradients and element partitioning that govern the resulting morphology. These microstructural refinements and orientation dispersions are intrinsic to the alloying process and serve as tangible indicators of the modified solidification pathways and phase transitions brought about by silver incorporation.

Figure 9c offers deeper insights into the grain boundary characteristics within the melting zone. The high average misorientation angle ($44.68^{\circ }$) suggests a dominance of high-angle grain boundaries (HAGBs). Notable misorientation peaks at $61.4^{\circ }$, $43.8^{\circ }$, and $57^{\circ }$ also indicate grain boundary stabilization due to Ag incorporation. Furthermore, the presence of a $4.2^{\circ }$ peak suggests the existence of low-angle grain boundaries (LAGBs), likely associated with subgrain structures formed due to rapid solidification. The coexistence of both LAGBs and HAGBs highlights the complex thermal history experienced during melting and resolidification.

Figure 9d illustrates the grain size distribution within the melting zone (MZ) of the Ti6Al4V–Ag specimen, revealing a refined microstructural profile characterized by a wide range of grain sizes. The distribution spans from approximately 0.13 µm to 3.44 µm, with an average grain width of 0.44 µm, which is notably finer than the 0.66 µm average grain size observed in the as-received alloy. This significant refinement can be attributed to the rapid cooling rates induced by laser processing, which hinder grain growth and promote the formation of ultrafine grains. The presence of such a narrow grain size distribution reflects the effectiveness of Ag incorporation in stabilizing grain boundaries and suppressing excessive coarsening during solidification.

Figure 9e,f reveal the pole figures of the α-phase (0001) and β-phase (001), respectively, in the melting zone (MZ) of the Ti6Al4V–Ag specimen. In Figure 9e, the α-phase pole figure exhibits a more scattered orientation distribution, indicating a weakened crystallographic texture. This suggests that the rapid solidification and high thermal gradients during laser processing disrupted the prior alignment of grains, leading to a more randomized orientation of the α-Ti plates. In contrast, Figure 4e, which depicts the α-phase in the as-received Ti6Al4V alloy, shows a stronger texture with clustered orientations along the rolling and transverse directions, characteristic of thermomechanically processed material. Similarly, Figure 9f presents the β-phase (001) pole figure of the Ag-modified zone, which remains weakly textured and diffusely oriented an outcome consistent with the rapid solidification behavior of the metastable β-phase. This is analogous to Figure 4f, where the β-phase of the base alloy also demonstrates minimal texture, reflecting its retention after high-temperature processing. These observations highlight the transformation of grain orientations due to laser surface alloying and underscore the influence of Ag in modifying texture intensity, particularly by promoting orientation scatter in the α-phase while preserving the inherently random nature of the β-phase.

**Figure 10:**
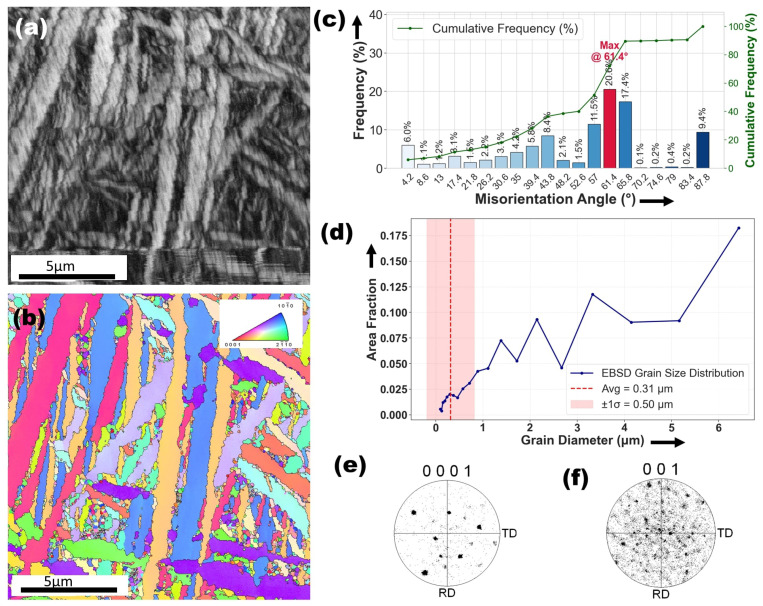
Microstructural and crystallographic analysis of the heat-affected zone (HAZ) of Ti6Al4V–Ag specimen. (**a**) BCC micrograph showing the transformed microstructure with elongated grains. (**b**) EBSD inverse pole figure (IPF) map revealing preferential grain orientation and retained lamellar structures. (**c**) misorientation angle distribution (**d**) grain size distribution plot. (**e**,**f**) pole figures for the α-phase (0001) and β-phase (001), with a strong preferred orientation in the α-phase.

Figure 10a captures the transformed microstructure within the heat-affected zone (HAZ) of the Ti6Al4V–Ag specimen, revealing elongated grains indicative of directional solidification or recrystallization triggered by the rapid thermal cycling during laser surface alloying. The corresponding IPF map in Figure 10b displays crystallographic orientations, with clear evidence of α and retained β phases, highlighting the presence of thermally driven phase transformations. This evolved microstructure contrasts with the as-received state depicted in Figure 4a, where equiaxed α grains and short-rod β-phase particles dominate, bounded predominantly by low-angle grain boundaries formed under recrystallization annealing. The strong texturing in the as-received α-phase, aligned along the rolling and transverse directions, becomes more scattered in the laser-modified zone, pointing to significant orientation redistribution. Such grain realignment and structural elongation are also reflected in Figure 5d–f, which visualizes the HAZ morphology and confirms the presence of martensitic plates and remnant β-phase particles. These observations suggest that the laser-induced thermal gradient not only facilitated martensitic transformation but also reorganized the texture of the alloy, thereby enhancing the microstructural complexity and promoting a refined phase structure.

Figure 10c the histogram provides a detailed analysis of the misorientation angle distribution in the heat-affected zone (HAZ) of the Ti6Al4V–Ag specimen. It reveals several critical insights into the microstructural transformations that have occurred due to thermal exposure. Significant peaks are observed around $57^{\circ }$, $61.4^{\circ }$, and $65.8^{\circ }$, with the highest frequency at $61.4^{\circ }$. Another notable peak occurs at $87.8^{\circ }$, indicating a presence of nearly perpendicular grain boundaries. There is a relatively low frequency of boundaries at misorientation angles below $20^{\circ }$. This suggests a dominance of high-angle boundaries, which are typically more favorable for grain boundary sliding and recrystallization processes. The average misorientation angle is approximately $51.7^{\circ }$, supporting the predominance of high-angle boundaries. The prevalence of high-angle boundaries (above $45^{\circ }$) indicates significant grain boundary energy, which can enhance properties like creep resistance and ductility.

Figure 10d presents the grain size distribution plot, which provides valuable insights into the microstructural characteristics of the HAZ in the Ti6Al4V–Ag specimen. It reveals a tendency for the area fraction to increase with larger grain sizes, especially for grains larger than approximately 3 μm. A peak in the area fraction is observed for the largest grain sizes, indicating a notable presence of larger grains. The smallest grain size is 0.1 μm, with an area fraction of 0.0093, while the largest grain size is 7.2 μm, with an area fraction of 0.1478. The area fraction generally increases with grain diameter, suggesting that larger grains occupy a significant portion of the microstructure. Notable peaks in the area fraction are observed for grains around 3 μm (area fraction 0.0872) and 7.2 μm (area fraction 0.1478). The average grain size is approximately 0.35 μm, with a standard deviation of 0.56 μm, indicating a broad distribution and considerable variability in grain sizes. This range and standard deviation point to heterogeneity, which may result in anisotropic material properties.

While larger grains dominate the microstructure, a significant fraction of fine grains (<1 μm) is also present, contributing to the overall grain size distribution. The dominance of larger grains could reduce material strength but enhance ductility and toughness. This suggests that the HAZ may be well-suited for applications requiring impact resistance or deformation. Conversely, the presence of fine grains can contribute to enhanced strength through grain boundary strengthening mechanisms, highlighting a balance in the material’s mechanical properties.

Pole Figure 10e,f show the distribution of specific crystallographic planes (e.g., (0001) for α-phase and (001) for β-phase) relative to a sample’s reference frame, such as the rolling direction (RD) and transverse direction (TD. Figure 10e: (0001) pole figure for α-phase shows strong texture with concentrated orientations, indicating a preferred alignment. Figure 10f: (001) pole figure for β-phase displays a more dispersed orientation, suggesting less pronounced texture. The preferred orientation is aligned along the rolling direction (RD) and transverse direction (TD), suggesting anisotropic properties. Concentrated points or clusters in a pole figure indicate that certain planes are preferentially oriented in specific directions. A strong texture is indicated by sharp, concentrated regions in the pole figure, showing that many grains share similar orientations.

**Figure 11:**
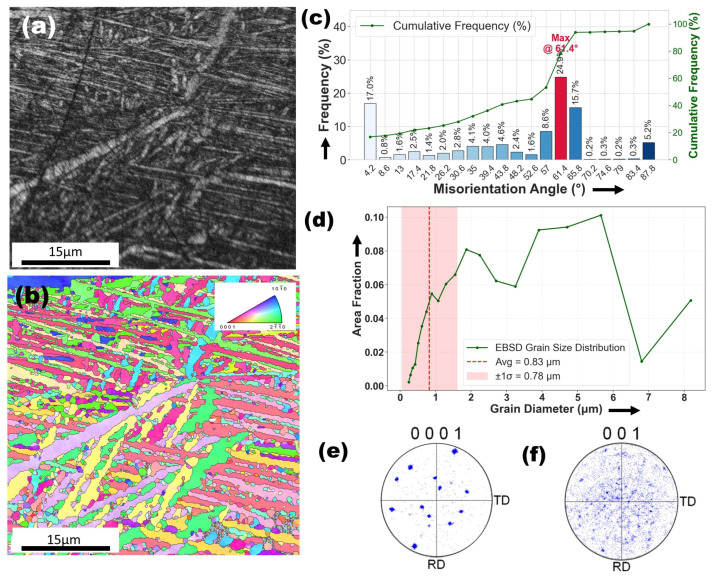
EBSD analysis of the melting zone (MZ) in the Ti6Al4V–Cu specimen: (**a**) band contrast (BC) image showing heterogeneous microstructure. (**b**) Inverse pole figure (IPF) map illustrating randomized grain orientations due to rapid solidification. (**c**) Misorientation angle distribution highlighting dominant high-angle grain boundaries (HAGBs) with peaks at 44°, 65.8°, and 87.8°. (**d**) Grain size distribution showing refined grains ranging from 0.24 to 8.19 µm. (**e**) Pole figure of the α-phase (0001) revealing moderate texture with clustered orientations. (**f**) Pole figure of the β-phase (001) displaying a weak and dispersed texture, indicating random orientation.

Figure 11a presents the BC image of the melting zone (MZ) in the Ti6Al4V–Cu specimen, revealing a heterogeneous microstructure characterized by bright, Cu-rich dendritic structures embedded within the Ti6Al4V matrix. The variation in contrast suggests compositional inhomogeneity, likely caused by partial copper segregation during rapid solidification. These microstructural features marked by refined dendritic morphology and the absence of porosity near the fusion boundaries are indicative of the rapid cooling dynamics inherent to the laser surface alloying (LSA) process. In contrast, the as-received Ti6Al4V alloy shown in Figure 4a exhibits a relatively homogeneous microstructure, comprising equiaxed α grains and uniformly distributed short-rod β-phase particles, without signs of dendritic growth or elemental segregation. The emergence of dendritic structures in the MZ, as further corroborated by SEM images in Figure 7a–c, reflects the transformation of the alloy’s solidification behavior due to localized melting and the presence of copper. Moreover, EDS analysis shown in Figure 8 confirms elevated copper concentrations specifically within the dendritic regions, while the surrounding matrix remains relatively depleted. This distinct elemental partitioning supports the formation of intermetallic compounds, most notably Ti_2_Cu, consistent with findings from previous studies on Cu-modified titanium alloys [14].

Figure 11c presents the misorientation angle analysis of the Ti6Al4V–Cu alloy, offering valuable insights into its grain boundary characteristics and microstructural evolution. The distribution of misorientation angles reveals significant peaks at $44^{\circ }$, $65.8^{\circ }$, and $87.8^{\circ }$ with the highest frequencies observed at $44^{\circ }$(24.86%) and $65.8^{\circ }$ (18.89%). These peaks indicate the dominance of high-angle grain boundaries (HAGBs), with a notable fraction of the microstructure exhibiting HAGBs. The average misorientation angle of $45.63^{\circ }$ further confirms this trend. The prevalence of HAGBs (>$15^{\circ }$) suggests that the laser surface alloying process has induced dynamic recrystallization and grain refinement, potentially improving the alloy’s mechanical strength and corrosion resistance. The misorientation angles around 60–56^∘^ may also point to the presence of twin boundaries, which could enhance the alloy’s toughness and ductility. Additionally, the peak at $87.8^{\circ }$ (8.18%) may be associated with specific crystallographic orientations linked to the Ti_2_Cu intermetallic phase or the β-phase grains inherited from the Ti6Al4V base alloy. In contrast to the as-received Ti6Al4V, which typically exhibits a dominance of low-angle grain boundaries (LAGBs, $15^{\circ }$) under certain conditions, the increased proportion of HAGBs in this study suggests that Cu addition plays a significant role in grain boundary formation and orientation evolution. This microstructural refinement could lead to enhanced hardness, wear resistance, and antibacterial properties, making the Ti6Al4V–Cu alloy more suitable for biomedical applications.

Figure 11d presents the grain size analysis of the Ti6Al4V–Cu alloy, confirming a significantly refined microstructure as a result of laser surface alloying. The grain sizes range from approximately 0.24 µm to 8.19 µm, with the majority falling within the 2.7 to 6.8 µm interval. The average grain size is measured at 0.83 µm, with a standard deviation of 0.78 µm, indicating a relatively uniform grain structure with moderate variability. The presence of ultrafine grains (0.24 to 0.8 µm) strongly suggests rapid solidification induced by the high thermal gradients and cooling rates characteristic of the laser process. This grain refinement is further enhanced by the addition of copper, which reduces grain boundary mobility and promotes the formation of the fine dendritic structures observed in the SEM images. When compared to the coarser and more heterogeneous grain structure of the base Ti6Al4V alloy (average grain size approximately 0.66 µm with a broader distribution), the microstructural refinement in the Cu-alloyed specimen indicates a clear modification of solidification behavior. Additionally, the formation of Ti_2_Cu intermetallic compounds may have contributed to this grain refinement by acting as nucleation sites and influencing local thermal gradients. The resulting fine-grain structure is expected to enhance mechanical performance, particularly in terms of strength and wear resistance, through the Hall–Petch effect. The presence of slightly larger grains (5 to 8 µm) in isolated regions may reflect localized variations in heat input or cooling rates during laser processing, further highlighting the complex thermal dynamics involved in microstructural evolution.

Figure 11e,f present the 0001 and 001 pole figures of the α-Ti and β-Ti phases, respectively, in the melting zone (MZ) of the Ti6Al4V–Cu alloy. The pole figure in Figure 11e shows moderately concentrated orientation clusters, indicating a preferred crystallographic orientation or weak basal texture in the α-phase. This suggests that during LSA, the rapid solidification and directional thermal gradients promoted the preferential growth of grains along specific crystallographic planes. Such behavior differs markedly from the as-received Ti6Al4V alloy shown in Figure 4e, where the pole figure reveals a stronger and more defined texture along the rolling and transverse directions due to prior thermomechanical processing. The observed reduction in texture intensity in the Cu-alloyed zone points to a more randomized grain reorientation, reflecting the disruption of rolling-induced texture by the laser process.

In Figure 11f, the β-phase (001) pole figure exhibits a dispersed and weak texture, indicating that the β-phase grains did not develop significant preferred orientation during rapid resolidification. This is consistent with both the as-received alloy’s β-phase pole figure (Figure 4f) and that of the Ag-modified MZ (Figure 9f), where the β-phase also showed a relatively random orientation distribution. This behavior supports the hypothesis that the β-phase, typically formed at high temperatures and rapidly transformed or retained during solidification, does not contribute significantly to overall texture evolution under rapid cooling conditions.

When compared to the Ti6Al4V–Ag melting zone (Figure 9e,f), the Ti6Al4V–Cu alloy shows more intense clustering in the α-phase pole figure, suggesting a relatively stronger basal texture. This could be attributed to Cu’s influence on solidification behavior, promoting oriented grain growth via solute redistribution and constitutional undercooling. In contrast, the Ag-modified MZ displayed a more diffuse α-phase texture, likely due to the different thermal conductivities and diffusion behaviors of Ag versus Cu.

Further comparison with the heat-affected zone (HAZ) of the Ag-modified specimen (Figure 10e,f) reveals that the α-phase in the Cu-modified MZ retains more texture than in the HAZ, where texture tends to weaken due to partial recrystallization and slower cooling rates. Similarly, while both Cu and Ag modified HAZs show clustered α-phase textures, the Cu-modified MZ demonstrates greater orientation coherence likely a result of stronger thermal gradients near the laser beam center.

**Figure 12:**
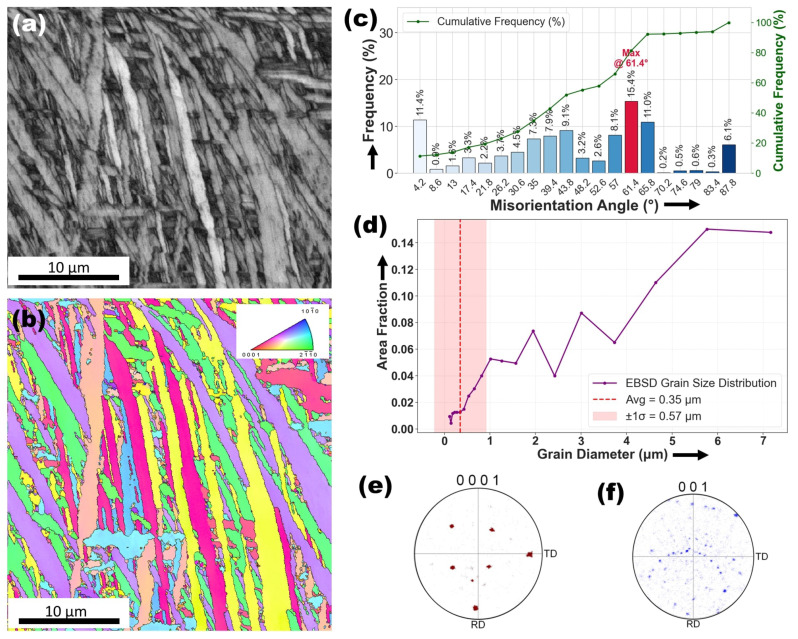
Microstructural and crystallographic analysis of the HAZ in the Ti6Al4V–Cu alloy specimen: (**a**) band contrast (BC) image showing elongated and irregular grain morphology; (**b**) inverse pole figure (IPF) map revealing strong crystallographic texture and diverse grain orientations; (**c**) misorientation angle distribution highlighting peaks at $4.2^{\circ }$, $61.4^{\circ }$, and $90^{\circ }$, indicating the presence of both low and high-angle grain boundaries; (**d**) grain size distribution showing a skew toward larger grains with an average size of 0.31 µm; (**e**,**f**) pole figures for the α-phase (0001) and β-phase (001), illustrating moderate texture in the α-phase and weak texture in the β-phase, respectively.

Figure 12a,b illustrate the microstructural and crystallographic characteristics of the heat-affected zone (HAZ) in the Ti6Al4V–Cu specimen following laser surface alloying. The band contrast (BC) image in Figure 12a reveals elongated and irregular grain structures, indicative of thermal recovery and the formation of martensitic plates during rapid cooling. The inverse pole figure (IPF) map in Figure 12b further confirms this transformation, showing a broad range of crystallographic orientations and a strong texture, which suggests directional solidification influenced by thermal gradients. These features closely align with the SEM observations in Figure 7e,f, where the HAZ exhibited acicular morphologies and densely packed, fine-twinned martensitic plates. This microstructural evolution supports the α → β → α martensitic transformation mechanism, driven by the rapid thermal cycle imposed by laser processing.

When compared to the as-received Ti6Al4V microstructure shown in Figure 4a,b characterized by equiaxed α grains and subgrains bounded by low-angle grain boundaries, the structure in Figure 12 shows significant transformation. The presence of elongated grains with increased orientation scatter and the prevalence of high-angle boundaries in the Cu-modified HAZ indicate a transition toward a more textured and recrystallized state. This evolution is quantitatively supported by the misorientation angle distribution in Figure 12c, which highlights prominent peaks at approximately $4.2^{\circ }$, 61.4°, and $90^{\circ }$. The peak at $4.2^{\circ }$ (with a high number fraction of 0.113856) indicates the presence of small-angle boundaries, likely related to subgrains or dislocation recovery. In contrast, the dominant peak at 61.4° (number fraction of 0.153659) and the additional peak at $90^{\circ }$ (0.0607595) point to a significant population of high-angle and possibly special boundaries, such as twin boundaries. The average misorientation angle of $44.52^{\circ }$ reflects a balanced distribution between low- and high-angle boundaries, suggesting the coexistence of deformation-induced structures and recrystallized grains. When compared with the HAZ of the Ag-alloyed specimen shown in Figure 10a,b, the Cu-modified HAZ exhibits a more elongated and textured grain structure, whereas the Ag-modified HAZ reveals relatively coarser grains and less directional elongation. This suggests that Cu addition promotes more pronounced orientation alignment and refinement due to its distinct thermal diffusivity and segregation behavior.

Figure 12d illustrates the grain size distribution within the HAZ, revealing that larger grains have a higher area fraction, particularly beyond 1 µm. The distribution is skewed towards larger grain sizes, with a peak in area fraction observed at the largest measured grains. Smaller grains, around 0.1 µm, exhibit relatively low area fractions, whereas the area fraction increases significantly with grain size, indicating the dominance of larger grains in the microstructure. The average grain size is 0.314486 microns, suggesting a moderate grain size, while the standard deviation of 0.504044 reflects considerable variability in the grain size distribution.

Figure 12e,f, representing the pole figures of the Ti6Al4V–Cu specimen’s heat-affected zone (HAZ), reveal a moderate degree of texture in the α-phase (0001) and a weaker, more dispersed texture in the β-phase (001). When compared to the as-received specimen in Figure 4e,f, where the α-phase exhibited strong clustering along the rolling and transverse directions due to prior thermomechanical processing, the Cu-modified HAZ shows a partially randomized orientation, reflecting the effects of thermal cycling and recrystallization during laser processing. In contrast, the β-phase in both cases retains a weak or near-random orientation. Compared to the Ag-modified HAZ in Figure 10e,f, which also displays strong preferred orientations in the α-phase and a relatively weak texture in the β-phase, the Cu-modified HAZ (Figure 12) shows slightly less intensity in the α-phase clusters, implying that Ag promotes stronger texture development than Cu under similar processing conditions. These differences underscore how Ag and Cu uniquely influence grain orientation: Ag tends to enhance alignment and promote anisotropy in the α-phase, while Cu induces a more distributed orientation due to its different thermal conductivity and diffusion behavior.

**Figure 13:**
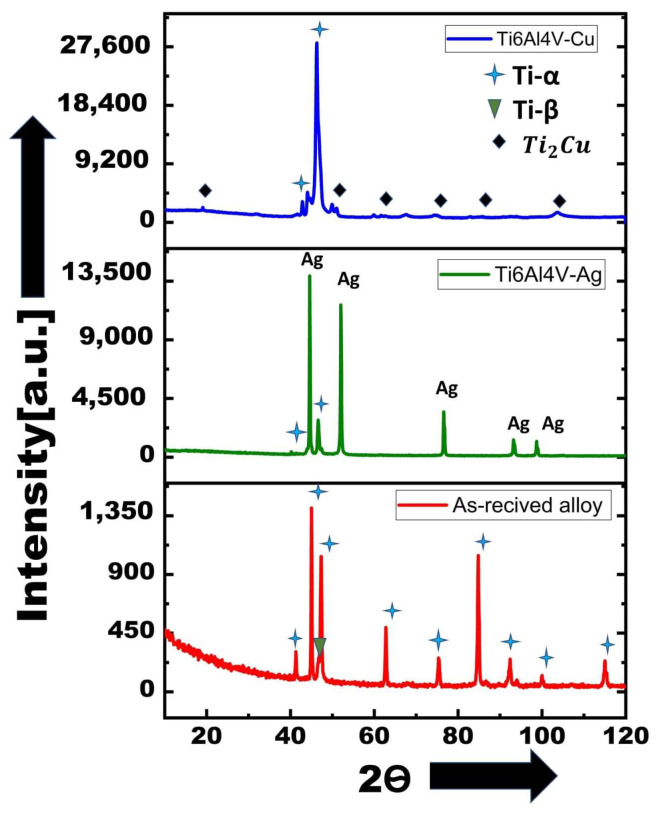
X-ray diffraction (XRD) patterns of as-received Ti6Al4V alloy (**bottom**), laser surface alloyed Ti6Al4V–Ag (**middle**), and Ti6Al4V–Cu (**top**). Ti-α and Ti- β phases are indexed with stars and inverted triangles, respectively. Intermetallic Ti_2_Cu peaks appear only in the Cu-modified sample, while metallic Ag reflections are detected exclusively in the Ag-treated sample.

Figure 13 presents the X-ray diffraction (XRD) patterns of the as-received Ti6Al4V, Ti6Al4V–Ag, and Ti6Al4V–Cu specimens, providing direct insights into phase evolution induced by laser surface alloying (LSA). The as-received sample exhibited characteristic peaks corresponding to α-Ti (hcp) and β-Ti (bcc) phases, confirming its dual-phase microstructure.

Upon Ag alloying, prominent peaks associated with metallic silver were observed at approximately $38\cdot 1^{\circ }$, $44\cdot 3^{\circ }$, and $77\cdot 4^{\circ }$, confirming the retention of elemental Ag within the modified surface. The Ti–α peaks remained dominant, with no evidence of new intermetallic compounds, indicating that Ag acted primarily as a surface modifier rather than a compound former.

In contrast, the Cu-modified sample displayed additional peaks at $36\cdot 2^{\circ }$, $61\cdot 2^{\circ }$, and $73\cdot 4^{\circ }$, which correspond to the Ti_2_Cu intermetallic phase, validating the EDS findings from Figure 8. These intermetallic peaks confirm that copper participates in compound formation during rapid solidification, leading to second-phase strengthening. Notably, the presence of both α-Ti and Ti_2_Cu suggests a heterogeneous solidification path with solute partitioning.

### 3.3. Hardness Comparison and Correlation with Microstructural Evolution

**Figure 14:**
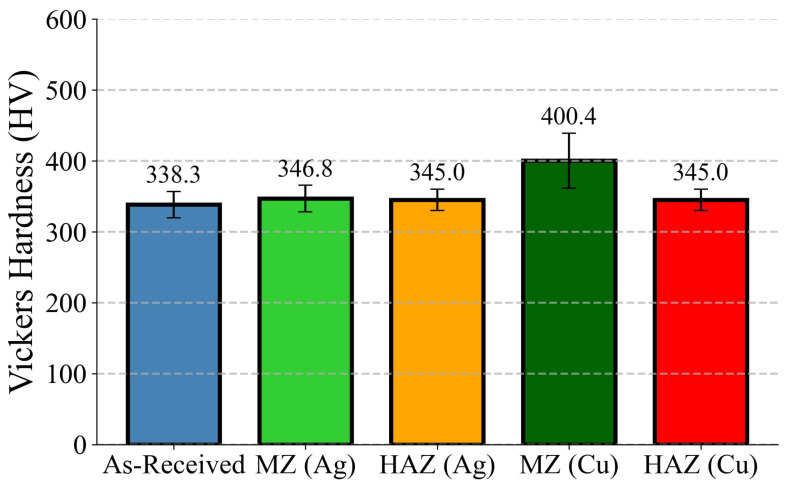
Vickers hardness of Ti6Al4V after laser surface alloying with Ag and Cu. MZ (Cu) shows the highest hardness (∼400.4), while all treated zones exceed the as-received value (∼338.3). Error bars indicate standard deviation.

Figure 14 presents the comparative Vickers hardness values across different regions of Ti6Al4V samples treated by laser surface alloying (LSA) with Ag and Cu. The as-received sample showed a baseline hardness of 338.3 HV, attributed to its recrystallized α–β microstructure dominated by equiaxed α grains and short-rod β-phase particles (Figure 4a). EBSD analysis (Figure 4b–d) further revealed a heterogeneous grain size distribution (average 0.66 µm) with predominantly low-angle boundaries, consistent with thermomechanically processed titanium alloys.

Upon Ag alloying, both the heat-affected zone (HAZ) and melt zone (MZ) exhibited modest hardness improvements 345.0 HV and 346.8 HV, respectively. SEM and EBSD characterizations (Figure 5 and Figure 9) indicated that the Ag modified MZ underwent substantial grain refinement (average grain size ∼0.44μm, Figure 9d) and developed dense martensitic plates alongside Ag-enriched dendrites (Figure 6). The texture became more randomized (Figure 9e,f), suggesting that rapid solidification disrupted prior grain alignment, reducing directional strengthening.

In contrast, the Cu-treated regions showed a significantly enhanced hardness, particularly in the MZ (400.4 HV), the highest among all regions. This can be directly linked to microstructural features captured in Figure 7, Figure 8 and Figure 11. The MZ of Ti6Al4V–Cu revealed a well-developed dendritic network enriched in copper, leading to the formation of hard intermetallics such as Ti_2_Cu (confirmed via EDS in Figure 8). These phases, in combination with a moderately refined grain size (∼0.83μm, Figure 11d), contribute to second phase strengthening and solute segregation hardening. Moreover, the EBSD derived misorientation distribution (Figure 11c) showed dominant HAGBs. The HAZ in Cu-modified samples also retained elevated hardness (345.0 HV) despite lacking the dendritic intermetallic network observed in the MZ. As shown in Figure 12, this zone exhibited elongated martensitic grains and higher grain boundary misorientation (Figure 12c), which are indicative of directional solidification and recrystallization effects induced by thermal gradients. Grain size distribution (Figure 12d) and pole figure analysis (Figure 12e,f) further confirmed moderate textural development and a heterogeneous grain structure, both contributing to the observed strengthening.

It is important to note that while the Ag-treated zones exhibited finer grains than their Cu counterparts, the absence of intermetallic reinforcement phases in Ag regions constrained the maximum achievable hardness. This observation aligns with prior reports [33,53] suggesting that martensitic transformation alone offers limited hardening compared to intermetallic strengthening mechanisms. The absence of such hard second phases in Ag-modified specimens limits the mechanical enhancement, supporting earlier findings by [28,39]. Beyond confirming these observations, the present work provides a unique contribution by systematically quantifying grain size distributions and misorientation angles in both the melt zone (MZ) and heat-affected zone (HAZ) after LSA. Ag incorporation promoted ultrafine martensitic plates with randomized texture and a high fraction of HAGBs, while Cu alloying induced dendritic growth, Ti_2_Cu precipitation, and solute segregation. These microstructural insights clarify how grain boundary evolution and intermetallic formation collectively govern hardness, offering guidance for optimizing load-bearing implants.

## 4. Conclusions

This study investigated the influence of laser surface alloying (LSA) with silver (Ag) and copper (Cu) on the microstructural evolution, phase constitution, and hardness of Ti6Al4V. The as-received alloy, with a baseline hardness of ∼338 HV, was significantly altered by the incorporation of Ag and Cu.

Ag-alloyed surfaces showed notable grain refinement in both the melt zone (average grain size ∼0.44 µm) and the heat-affected zone (∼0.35 µm). SEM revealed dendritic or cellular structures enriched in Ag together with fine martensitic plates. XRD confirmed the retention of metallic Ag without evidence of new Ag–Ti intermetallics, suggesting that Ag primarily segregated rather than forming compounds. Consequently, the hardness of the Ag-modified regions increased modestly, reaching ∼347 HV in the melt zone and ∼345 HV in the heat-affected zone.

Cu-modified surfaces, in contrast, developed dendritic networks and refined grains (melt zone ∼0.83 µm; heat-affected zone ∼0.31 µm). Phase analysis revealed the presence of Ti_2_Cu intermetallics alongside $\alpha^{\prime }$ martensite, which contributed to a more pronounced hardness increase, with the melt zone reaching ∼400 HV and the heat-affected zone maintaining ∼345 HV, both above the base alloy.

Both Ag and Cu alloying improved the microstructure of Ti6Al4V, albeit through different mechanisms. Ag promoted ultrafine martensitic structures without forming new intermetallics, leading to moderate hardness gains, whereas Cu induced Ti_2_Cu precipitation in addition to martensite, producing a higher hardness response. These results indicate that Cu-modified zones exhibited greater hardness than Ag-modified zones, suggesting a stronger surface strengthening effect under the tested conditions. However, it should be emphasized that hardness alone does not provide a complete measure of mechanical performance. Further studies involving tensile, fatigue, toughness, and tribological testing are needed to comprehensively evaluate and compare the strengthening effects of Ag and Cu alloying. 

## Data Availability

The data presented in this study are available on request from the corresponding author.
